# Obinutuzumab (GA101) vs. rituximab significantly enhances cell death, antibody-dependent cytotoxicity and improves overall survival against CD20+ primary mediastinal B-cell lymphoma (PMBL) in a xenograft NOD-scid IL2Rgnull (NSG) mouse model: a potential targeted agent in the treatment of PMBL

**DOI:** 10.18632/oncotarget.27691

**Published:** 2020-08-11

**Authors:** Yaya Chu, Aradhana Awasthi, Sanghoon Lee, Dina Edani, Changhong Yin, Jessica Hochberg, Tishi Shah, Tae-Hoon Chung, Janet Ayello, Carmella van de Ven, Christian Klein, Dean Lee, Mitchell S. Cairo

**Affiliations:** ^1^Department of Pediatrics, New York Medical College, Valhalla, NY, USA; ^2^Department of Cell Biology & Anatomy, New York Medical College, Valhalla, NY, USA; ^3^Department of Microbiology & Immunology, New York Medical College, Valhalla, NY, USA; ^4^Department of Medicine, New York Medical College, Valhalla, NY, USA; ^5^Department of Pathology, New York Medical College, Valhalla, NY, USA; ^6^Cancer Science Institute of Singapore, National University of Singapore, Singapore; ^7^Roche Pharmaceutical Research & Early Development, Roche Innovation Center, Zurich, Switzerland; ^8^Department of Pediatrics, Nationwide Children’s Hospital, Columbus, Ohio, USA; ^*^Co-first authors

**Keywords:** Obinutuzumab, rituximab, survival, primary mediastinal large B-cell lymphoma, antibody-dependent cellular cytotoxicity

## Abstract

Primary mediastinal large B-cell lymphoma (PMBL), a distinct mature B-cell lymphoma, expresses CD20 and has recently been successfully treated with the combination of a type I anti-CD20 monoclonal antibody, rituximab, with multiple combination chemotherapy regimens. Obinutuzumab is a glycoengineered type II anti-CD20 monoclonal antibody (mAb), recognizing a unique CD20 extracellular membrane epitope with enhanced antibody dependent cellular cytotoxicity (ADCC) vs rituximab. We hypothesize that obinutuzumab vs rituximab will significantly enhance *in-vitro* and *in-vivo* cytotoxicity against PMBL. PMBL cells were treated with equal dose of obinutuzumab and rituximab for 24 hours (1–100 μg/ml). ADCC were performed with *ex-vivo* expanded natural killer cells at 10:1 E: T ratio. Mice were xenografted with intravenous injections of luciferase expressing Karpas1106P cells and treated every 7 days for 8 weeks. Tumor burden was monitored by IVIS spectrum system. Compared with rituximab, obinutuzumab significantly inhibited PMBL cell proliferation (*p* = 0.01), promoted apoptosis (*p* = 0.05) and enhanced ADCC (*p* = 0.0002) against PMBL. Similarly, in PMBL xenografted NOD scid gamma mice, obinutuzumab significantly enhanced survival than rituximab when treated with equal doses (*p* = 0.05). Taken together our results suggest that obinutuzumab significantly enhanced natural killer cytotoxicity, reduced PMBL proliferation and prolonged the overall survival in humanized PMBL xenografted NOD scid gamma mice.

## INTRODUCTION

Primary mediastinal large B-cell lymphoma (PMBL) is a rare form of non-Hodgkin lymphoma representing < 5% of mature B-cell NHL (B-NHL) [1, 2]. The immunophenotypic features and molecular characteristics distinguish PMBL from other types of aggressive B-NHL and large B cell lymphoma [3, 4]. PMBL shares one third of its genes with Hodgkin lymphoma [3]. PMBL is classified as midway in the biologic spectrum between diffuse large B-cell lymphoma (DLBCL) and classical Hodgkin lymphoma [3] with overexpression of genes in signaling pathways of NF-kB and Janus kinase (JAK)/signal transducer and transcription (STAT), high expression of CD20, downregulation of major histocompatibility class I and II molecules and overexpression of programmed death ligands [2, 5–9].

Obinutuzumab is a glycoengineered humanized monoclonal antibody (mAb) recognizing a unique CD20 type II epitope [10] with significant antibody dependent cellular cytotoxicity (ADCC) enhanced as compared to rituximab *in-vitro* and *in-vivo* [11–14]. The anti-tumor effects of obinutuzumab alone or in combination with other agents were further investigated in clinical trials. The safety and efficacy of obinutuzumab was compared with rituximab in relapsed indolent lymphoma in the randomized phase II trial (GAUSS) (NCT00576758) [15]. Among patients with follicular lymphoma (FL), obinutuzumab demonstrated a higher overall response rate than rituximab (44.6% v 33.3%; *P* = .08) but with no difference in progression-free survival (PFS) between the two arms [15]. The phase III GALLIUM trial (NCT01332968) and GADOLIN trial (NCT01059630) were conducted to treat previously untreated FL patients or patients with rituximab-refractory indolent non-Hodgkin lymphoma utilizing obinutuzumab combined with chemotherapy [16, 17]. Obinutuzumab-based therapy significantly reduced the risk of progression or death and prolonged overall survival (OS) as compared to rituximab-based therapy or chemotherapy [16, 17]. Obinutuzumab plus chlorambucil prolonged the OS or PFS and resulted in higher rates of complete response in patients with chronic lymphocytic leukemia (CLL) or coexisting conditions as compared to chlorambucil alone, or rituximab plus chlorambucil, respectively in the CLL11 clinical trial (NCT01010061b) [18]. Furthermore, the phase III iLLUMINATE trial (NCT02264574) demonstrated that obinutuzumab plus Ibrutinib is an efficacious combination therapy for previously untreated patients with CLL or small lymphocytic lymphoma [19]. Based on these exciting results, obinutuzumab in combination with chemotherapy has been approved for the treatment of untreated and rituximab refractory FL [16, 17] and CLL [19]. Unfortunately, the clinical results of obinutuzumab for patients with DLBCL were not promising. Obinutuzumab was not superior to rituximab when combined with chemotherapies in patients with DLBCL shown in the phase III GOYA trial (NCT01287741) and the GAINED trial (NCT01659099) [20–22]. Additionally, the pre-clinical and clinical efficacy of obinutuzumab compared to rituximab in patients with PMBL is currently unknown.

We hypothesize that obinutuzumab would be a superior anti-CD20 antibody in the treatment of PMBL by inducing targeted programmed cell death and enhancing immune cell mediated ADCC compared to rituximab. In this study, we report the *in-vitro* and *in-vivo* efficacy of obinutuzumab against PMBL cell lines and in human PMBL xenografted immunodeficient NOD scid gamma (NSG) mouse model compared to rituximab.

## RESULTS

### Expression of CD20 mRNA and protein in obinutuzumab treated PMBL

**Figure 1:**
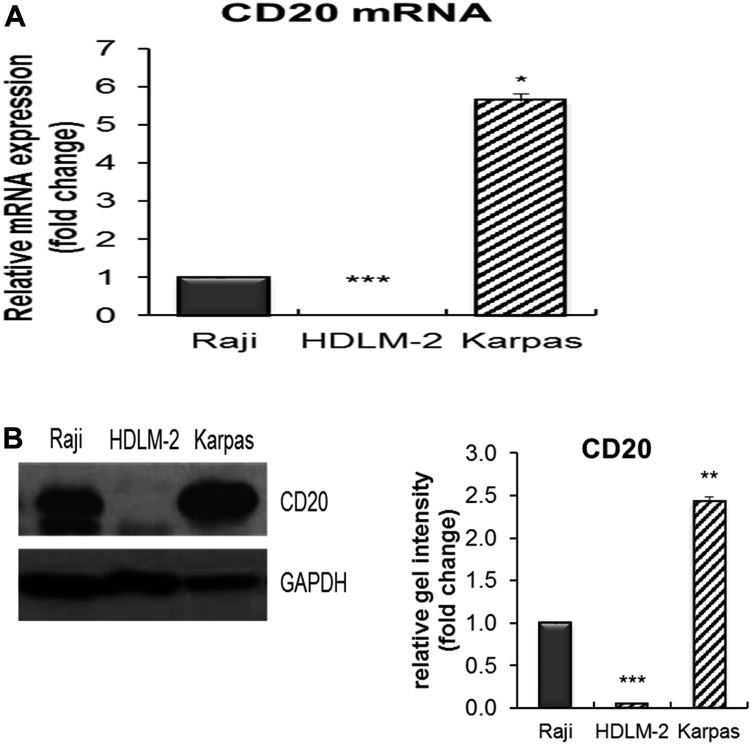
The expression of CD20 mRNA and protein in Karpas-1106 PMBL cells. (**A**) The CD20 mRNA, (**B** left) protein expression and (B right) its band intensity in Karpas-1106P PMBL cell line by qRT-PCR and immunoblotting compared to Raji (BL) and HDLM-2 (HL) cells as controls. Data are represented as the mean ± SD, ^*^ *p* < 0.01; ^**^ *p* < 0.001; ^***^ *p* < 0.00005 (*N* = 3).

CD20 mRNA and protein expression in Karpas-1106P were measured by real time quantitative reverse transcription polymerase chain reaction and immunoblotting prior to any anti-CD20 treatment. Karpas-1106P showed a significant increase in the expression of both CD20 mRNA and protein (Figure 1A and 1B) compared to Burkitt lymphoma (BL) (Raji) and Hodgkin lymphoma (HDLM-2) cell lines.

### Significant decrease of cell viability in obinutuzumab treated PMBL

**Figure 2:**
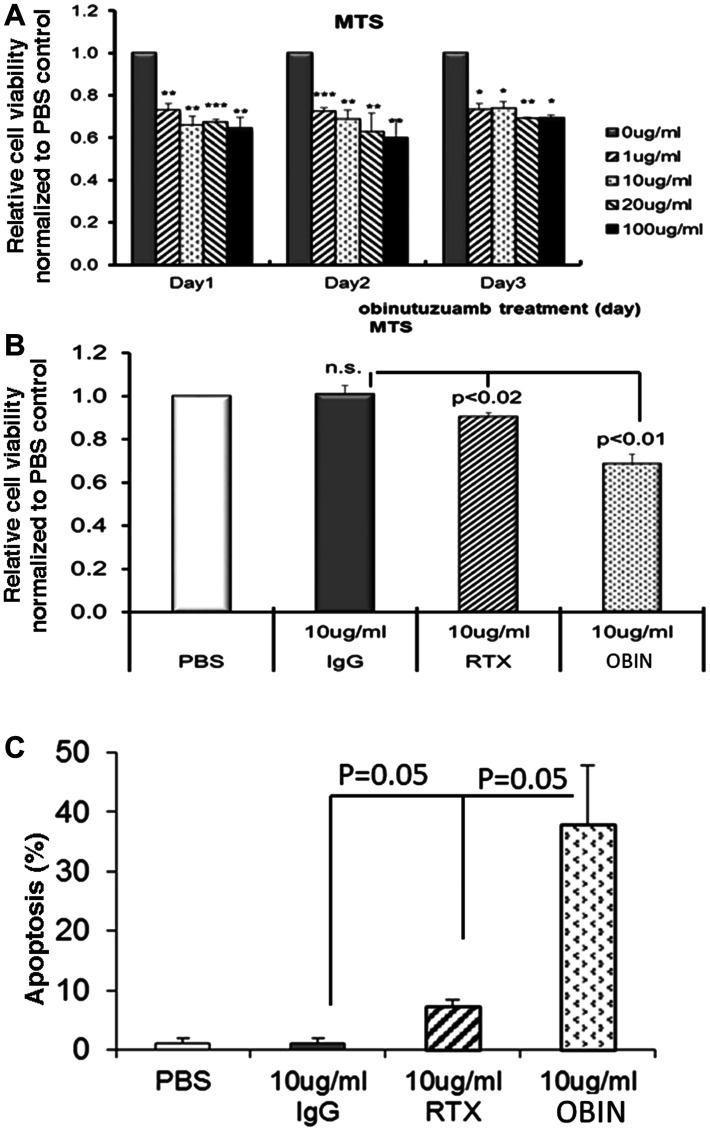
Cell proliferation and apoptosis activity in obinutuzumab treated Karpas-1106P PMBL cells. (**A**) Cells were plated (1 × 10^5^) into 48 well plates and cell proliferation was measured every 48 hours via MTS assay following obinutuzumab treatment (0, 1, 10, 20 and 100 μg/ml) in Karpas-1106P PMBL cells. (**B**) Significant inhibition in cell proliferation in obinutuzumab (*p* < 0.01) and rituximab (*p* < 0.02) treated Karpas-1106P cells compared to PBS control. Data are represented as the mean ± SD, ^*^ *p* < 0.05; ^**^ *p* < 0.01; ^***^ *p* < 0.001. (**C**) Apoptosis assays were performed by flow cytometric analysis using FITC-Annexin V/PI staining in obinutuzumab (10 ug/ml) treated Karpas-1106P PMBL cells compared to PBS control. Significant increase of apoptosis in obinutuzumab (*p* = 0.05) and rituximab (*p* = 0.05) treated Karpas-1106P cells compared to PBS control. Data are represented as the mean ± SD of triplicates (paired *t* test) (*N* = 3).

Karpas-1106P cells were treated with obinutuzumab, rituximab and IgG-isotype and viable cells were quantified by MTS assay. There was a significant decrease of viable cell number in the obinutuzumab-treated Karpas-1106P compared to control cells at each day regardless of the dose of obinutuzumab from 1–100 μg/ml (Figure 2A). Specifically, we observed significant reduction of viable cells with > 27% (*p* < 0.01), > 34% (*p* < 0.01), > 33% (*p* < 0.001) and > 35% (*p* < 0.01) in 1, 10, 20 and 100 μg/ml obinutuzumab treated Karpas-1106P cells for 24 hours and significantly reduction with > 27% (*p* < 0.001), > 31% (*p* < 0.01), > 37% (*p* < 0.01) and > 40% (*p* < 0.01) at 48 hours, respectively. In addition, we also observed significant reduction of viable Karpas-1106P cells by obinutuzumab at 1 (> 27%, *p* < 0.05), 10 (< 26%, *p* < 0.05), 20 (< 31%, *p* < 0.01), and 100 μg/ml (< 30%, 0.05) for 72 hours treatment compared to controls.

We further compared the cell viability of PMBL cells treated with obinutuzumab vs. rituximab. We found that the viability of Karpas-1106P cells was significantly reduced in the samples incubated with Obinutuzumab (10 μg/ml) compared to the samples incubated with rituximab (10 μg/ml) (viability reduction: 29.5% ± 4.60 vs. 4.4% ± 1.29, *p* < 0.01) at 48 hours (Figure 2B).

### Significant increase in obinutuzumab-induced cell death in PMBL

Next, we compared the cell death in PMBL induced by 10 μg/ml obinutuzumab compared to 10 μg/ml rituximab at 48 hours treatment. Cell apoptosis was significantly enhanced after obinutuzumab treatment compared to rituximab at similar dose concentration (37.8% ± 10.096 vs. 7.16% ± 2.969 (*p* < 0.05) (Figure 2C).

### Effect of ADCC by obinutuzumab vs. rituximab in combination with expanded natural killer (NK) cells against PMBL

**Figure 3:**
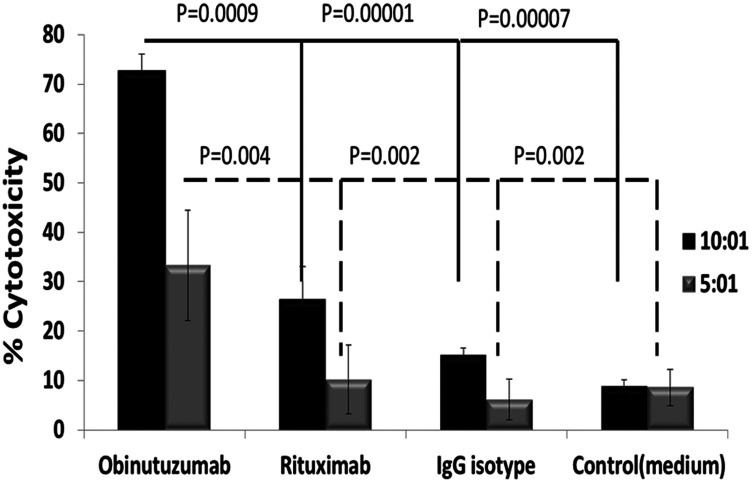
Obinutuzumab is significantly more effective in inducing NK cell mediated ADCC activity compared to rituximab. Karpas-1106P cells were treated with obinutuzumab, rituximab and IgG isotype control (10 μg/ml) and incubated with expanded and activated NK cells for additional 4 hours at 37°C at a 5:1 & 10:1 effector: targets ratio. Cell lysis was determined by manufacturer’s protocol using DELFIA cell cytotoxicity assays. Data shown are mean ± SD (*n* = 4) obinutuzumab vs rituximab, (*p* = 0.0009 and *p* = 0.004).

We observed that obinutuzumab + expanded NK cells had significantly increased ADCC vs. rituximab + expanded NK with same antibody doses (10 μg/ml) against PMBL cells; 73.4 ± 3.4 vs 38.3 ± 9.2% (*p* = 0.0009) at 10:1 and 33.2 ± 11.1% vs. 11.6 ±.3.7% (*p* = 0.004) at E: T ratio 5:1 (*n* = 4) (Figure 3).

### Obinutuzumab effects on the STAT6/NF-kB/PI3K/AKT pathway

**Figure 4:**
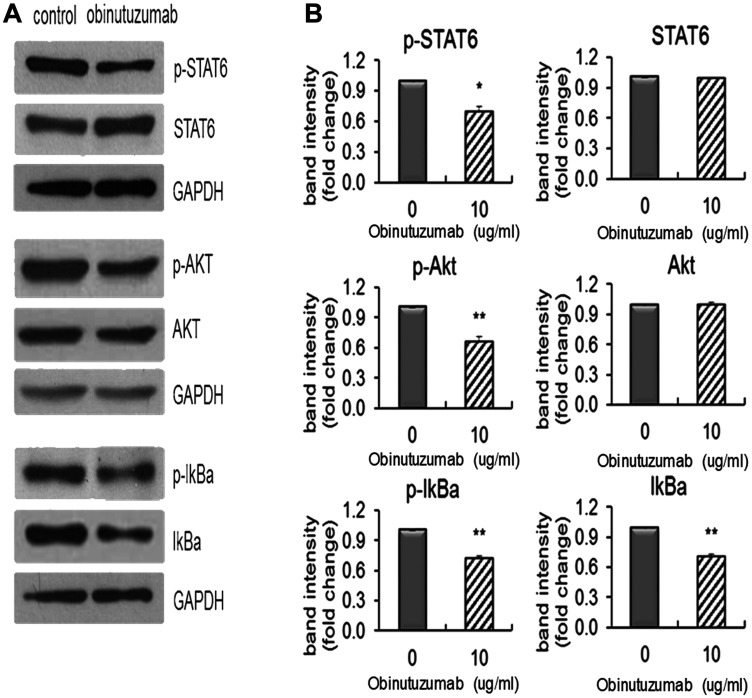
Obinutuzumab effect on the STAT6 and NFkB/PI3K pathways in PMBL cells. Karpas-1106P PMBL cells were treated with or without obinutuzumab (10 μg/ml) for 48 hours and analyzed by (**A**) western blotting, and (**B**) the intensity of the bands was quantified by Image J. Data are represented as the mean ± SD. ^*^ *p* < 0.05; ^**^ *p* < 0.005 (*N* = 3).

Constitutive STAT6 activation is identified as a key feature of PMBL from other lymphoma and the NF-κB signaling pathway and the PI3K/AKT pathway are also reported to be activated in PMBL [2, 23]. Based on this known information, we firstly examined if obinutuzumab could potentially inhibit these pathways by decreasing the phosphorylations of some key members of these pathways. Karpas-1106P cells were treated with obinutuzumab (10 μg/ml) for 48 hours and the phosphorylation levels of AKT, STAT6 and IκBα were measured by Western blotting. There was a significant decrease of p-STAT6 (treated vs control: 0.725 ± 0.0065, *p* < 0.05) in the JAK/STAT pathway, p-AKT (treated vs control: 0.69 ± 0.0028, *p* < 0.005) in the PI3K/AKT pathway and p-IκBα (treated vs control: 0.706 ± 0.009, *p* < 0.005) in the NF-κB pathway in obinutuzumab treated Karpas 1106P cells compared to PBS controls (Figure 4A and 4B). There were no significant differences of total STAT6 and AKT proteins between the treated and the controls (treated vs control: 0.978 ± 0.0071, *p* > 0.05; 0.976 ± 0.011, *p* > 0.05; respectively) (Figure 4B). Total IκBα protein level was significantly reduced in obinutuzumab treated Karpas-1106P cells compared to PBS controls (treated vs control: 0.682 ± 0.0066, *p* < 0.005) (Figure 4B).

### Gene expression changes in obinutuzumab treated PMBL cells

**Figure 5:**
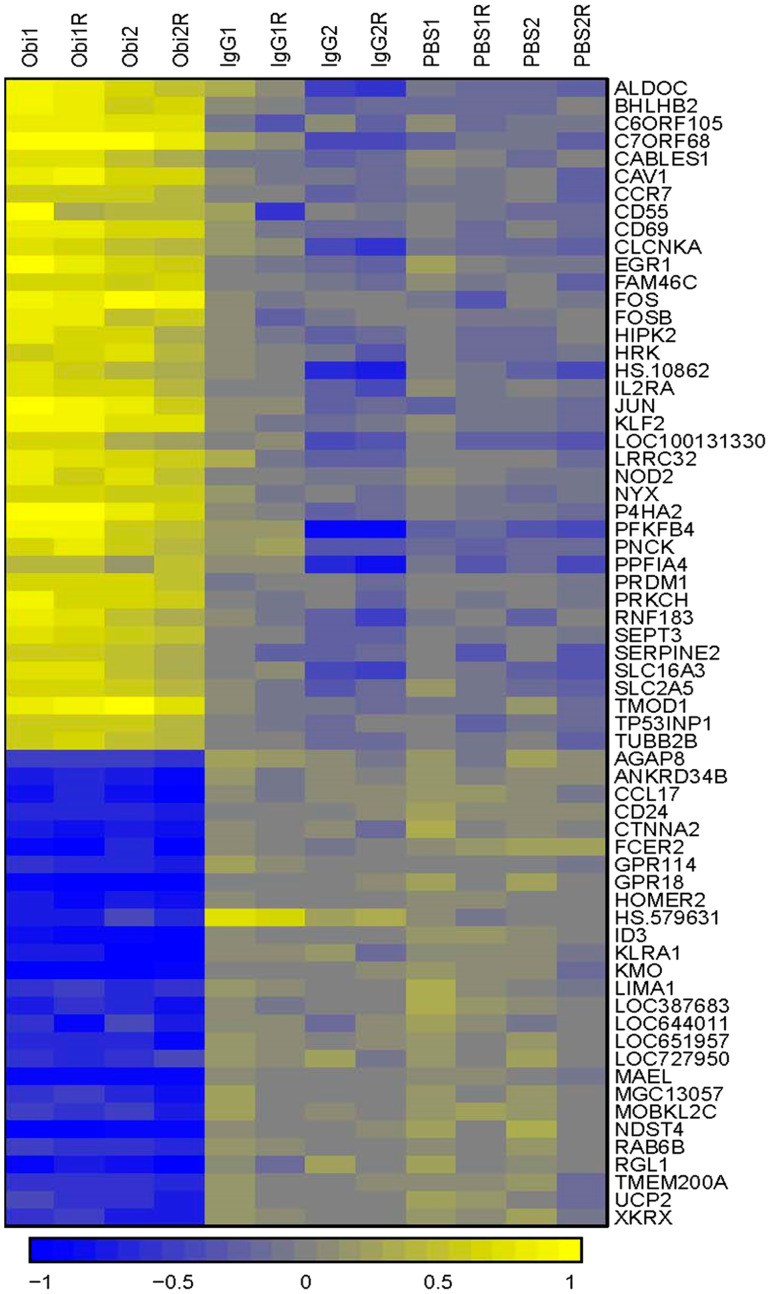
Hierarchical clustering comparing gene expression patterns between obinutuzumab and human IgG or PBS treated cells. A total of 65 differentially expressed genes were identified by gene expression profiling (> 1.5-fold) in obinutuzumab treated Karpas-1106P PMBL cells compared to PBS and IgG controls. Yellow represents increase expression and blue decreased expression, according to the scale. (*N* = 3).

To identify other biological processes affected by obinutuzumab, we investigated gene expression changes in obinutuzumab treated PMBL cells. Genomics employing Illumina microarray technology in obinutuzumab treated Karpas-1106P PMBL cells were compared to PBS or IgG isotype control. A total of 65 differentially expressed genes (0.3% of all genes) were identified (>1.5-fold) from 21,381 human genes (Figure 5). Among the 65 filtered genes, 38 genes (58.5%) were up-regulated and 27 (41.5%) genes including ID3 and RAB6B were down-regulated. When we performed gene set enrichment analysis (GSEA) to identify the biological processes affected by the obinutuzumab treatment, we observed 19 gene sets were enriched to the obinutuzumab treatment while 8 were enriched to the IgG treatment (Supplementary Table 1) and 9 were enriched after PBS treatment (Supplementary Table 2), respectively. Gene sets enriched in the obinutuzumab treatment group included IL6-JAK-STAT6 signaling, Hedgehog signaling, interferon gamma signaling, apoptosis and MTORc1 signaling pathways (Supplementary Tables 1 and 2).

### Effect of obinutuzumab on survival in human PMBL xenografted NSG mice

**Figure 6:**
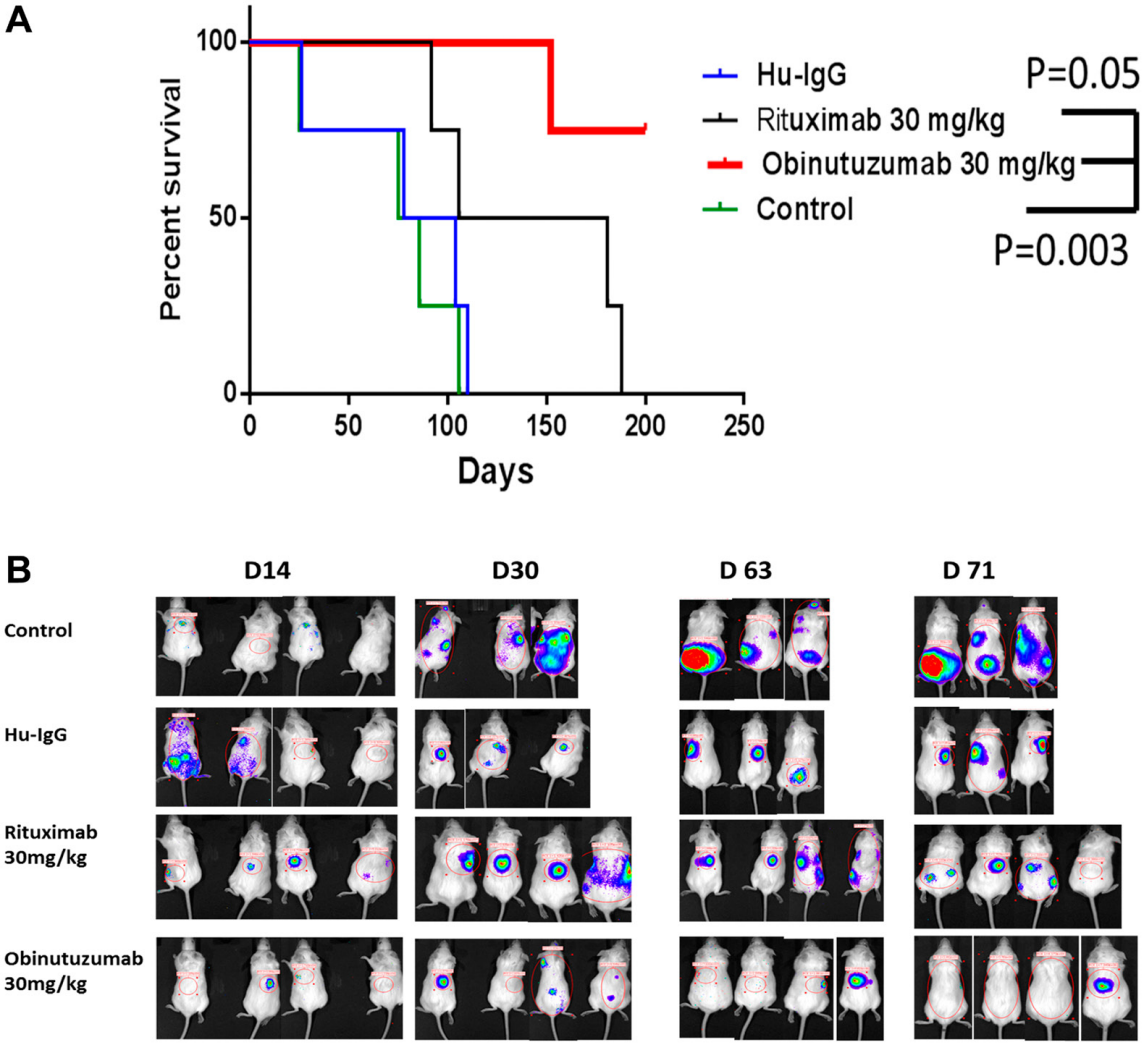
Effect of Obinutuzumab vs. Rituximab in PMBL NSG xenograft *in-vivo*. NSG mice were injected intravenously with human Karpas-1106P Luc+. Survival rates were analyzed following PBS, IgG, obinutuzumab, or rituximab treatment (30 mg/kg) once a week till 8 weeks. (**A**) The Karpas1106p-Luc xenografted NSG mice were observed until day 250. The Kaplan–Meier survival curves were generated following therapy initiation until mice death or mice sacrifice. Comparison of survival between groups is shown. Obinutuzumab significantly extended the survival of Karpas1106p-Luc mice compared to the group treated with rituximab (*p* < 0.05) or with PBS/IgG (*p* < 0.003). (**B**) Representative bioluminescence imaging of Karpas1106p-Luc xenografted NSG mice are shown at day 14, day 30, day 63 and day 71 after implantation of Karpas1106p-Luc cells in different treatment groups.

We observed that obinutuzumab (30 mg/kg) treated Karpas-1106P PMBL cells xenografted NSG mice had significantly increased prolonged survival time with a median of 200 days compared to rituximab (143.5 days, *p* = 0.05), control (80 days, *p* = 0.003) and hu-IgG (91 days) (Figure 6A). The obinutuzumab treated group significantly reduced tumor burden compared to rituximab treated group (day 63, *p* = 0.03) (Figure 6B).

## DISCUSSION

We have demonstrated significant *in-vivo* and *in-vitro* effects of obinutuzumab against PMBL. Specifically, we describe superior efficacy of obinutuzumab vs. rituximab (10 μg/ml) against Karpas 1106-P PMBL cells, including significantly decreased cell viability, increased cell death and enhanced ADCC. In addition, PMBL xenografted NSG mice treated with obinutuzumab showed a significant increase in survival compared with rituximab.

Obinutuzumab has been reported to have increased cytotoxicity causing B-cell depletion with greater activity against CD20+ tumor cells resulting in improved overall survival [11, 24]. Treatment in adults with relapsed indolent CD20+ B-NHL has demonstrated a higher overall response rate compared to rituximab [15]. As mentioned earlier, studies of obinutuzumab vs rituximab in patients with newly diagnosed or aggressive DLBCL in the upfront setting have not shown the same survival advantage [21, 22]. The reasons for the lack of advantage with obinutuzumab in DLBCL were unclear. This could be the result of differences in biologic and clinical profiles as well as differences in the immune microenvironment [22].

In contrast to the Type I anti-CD20 antibody rituximab, the tumor-killing mechanisms of obinutuzumab are reported to associate with enhanced direct cell death and ADCC, but reduced CDC [25]. Treatment of Karpas-1106P PMBL cells with obinutuzumab showed significant decreased cell viability irrespective of administered dose (Figure 2). This report is also consistent with increased cell death by increased ADCC in BL and pre B-cell acute lymphoblastic leukemia cell lines [11], and by increased activation of polymorphonuclear neutrophils resulting in increase in phagocytosis in chronic lymphocytic lymphoma cell lines [26]. Consistently with previous reports that obinutuzumab induced superior ADCC with effector cells using peripheral blood mononuclear cells [24], we found that obinutuzumab significantly enhanced ADCC mediated by *ex vivo* expanded NK cells (Figure 3). Constitutive activation of STAT6 pathway in PMBL has been previously reported by other investigators [27]. Differential regulation of AKT phosphorylation by obinutuzumab has also been reported in a 3D NHL model [28, 29]. STAT6, NF-κB and PI3K/AKT pathways were reported to be activated in PMBL [2, 23]. Our group recently demonstrated the difference in differential phosphorylation of 978 unique phosphoproteins between rituximab sensitive and resistant BL cell lines after obinutuzumab vs. rituximab treatments [12]. In this study, we found that obinutuzumab significantly reduces the phosphorylations of STAT6, AKT and IκBα in PMBL compared to controls (Figure 4). The treatment of a PMBL cell line with obinutuzumab differentially phosphorylated the PI3K/AKT pathway. This, in turn, affected phospho- IκBα and phospho-STAT6 signaling and NF-kB, JAK/STAT signaling pathways. Our results indicate that obinutuzumab inhibits PMBL proliferation partially by reducing the activation of the PI3K/AKT, NF-kB, JAK/STAT pathways.

Obinutuzumab enhances ADCC through several mechanisms. The prominent one is involved in enhancing the binding affinity to the FcγRIII receptor on immune effector cells such as NK cells through the post-translational glycoengineering [25, 30]. The second mechanism is that obinutuzumab overcomes the inhibition from inhibitory killer cell Ig-like receptors (KIRs) on NK cells [31]. Another mechanism is related to CD32B mediated internalization. CD32B is the inhibitor Fcγ receptor IIB (FcγRIIB) with low-affinity for IgG [32]. It promotes rituximab internalization from B cells and abrogates ADCC, which contributes to rituximab resistance and is correlated with poor clinical responsiveness [33, 34]. Obinutuzumab was found to be less sensitive to the internalization, partially due to their ineffectiveness to redistribute CD20 into lipid rafts [35] and less ability to interact with and activate CD32B [36]. Obinutuzumab significantly enhanced expanded NK mediated ADCC than rituximab (Figure 3) possibly through all of the mechanisms.

Gene expression analysis uncovered very small number of genes (0.3%) exhibiting differential expression between obinutuzumab treated and control treated cell lines. GSEA clearly revealed the upregulation of genes related to apoptosis and mitogen-activated protein kinase signaling pathway and protracted downregulation of genes related to cell cycle and cell proliferation including ID3 and RAB6B though the reason for this is not well understood [28, 37, 38].

We demonstrated that obinutuzumab showed improved survival and inhibition of tumor progression in PMBL xenografted NSG mice. This data is consistent with superior activity of obinutuzumab in other B-cell lymphoma xenografts when used alone or in combination with other agents [1, 39–43].

In conclusion, this study reveals that using obinutuzumab as a single agent is significantly more effective in PMBL cells both *in-vitro* and *in-vivo* utilizing PMBL xenografted NSG mice compared to equal doses of rituximab. This novel anti-CD20 type II antibody should be considered as an alternative treatment for PMBL utilizing chemoimmunotherapy regimens.

## MATERIALS AND METHODS

### Reagents and antibodies

PMBL Karpas-1106P cells (DSMZ, Germany) were cultured in RPMI 1640 with 20% FBS [24]. Obinutuzumab was generously provided by Hoffman LaRoche (Basel, Switzerland). Antibodies specific for phospho-protein kinase B, phospho-IκBα, phosphor-Stat6, AKT, IκBα and Stat6, were purchased from Cell Signaling Technology (Cambridge, MA & Santa Cruz, CA, USA).

### MTS assay

Karpas-1106P cells (5.0 × 10^5^) were treated with obinutuzumab at increasing dose concentrations (0, 1, 10, 20 and 100 μg/ml) for three days. Cell viability was measured by CellTiter 96 AQueous one solution cell proliferation assay (Pormega, Madison, WI, USA) [44] and measured by a multifilter plate reader (Molecular Device, CA, USA) at OD490. The obinutuzumab dose of 10 μg/ml was then compared to equal dosing of rituximab (10 μg/ml).

### Immunoblotting

Whole-cell extracts were resolved on sodium dodecyl sulfate polyacrylamide gel electrophoresis, transferred to nitrocellulose membrane, probed with appropriate antibodies and blocked in 5% milk for 1 hour, subsequently incubated with primary antibodies at 4°C for overnight and developed using Enhanced Chemiluminescence Reagent (GE Healthcare Bio-Sciences, Piscataway, NJ, USA) [44]. Band intensities on sodium dodecyl sulfate polyacrylamide gel electrophoresis were measured using ImageJ software.

### Quantitative reverse-transcription polymerase chain reaction

Total RNA was prepared using Trizol reagent (Invitrogen) according to the manufacturer’s manual. The cDNA was then synthesized using qScript™ cDNA Synthesis Kit (Quantas). Relative quantification (ddCt) of mRNA expression was determined by normalizing to the housekeeping gene (GAPDH).

### Cell apoptosis assays

Cell death was assayed by flow cytometric analysis (Beckman Coulter, Brea, CA, USA) of Annexin V, and propidium iodide (BD Biosciences, San Jose, CA, USA). 3 × 10^5^ cells were either untreated (control) or treated with 10 μg/ml of obinutuzumab, rituximab or human-immunoglobulin G for 24 to 72 hours. Annexin V/PI staining was performed according to the manufacturer instructions.

### NK cell isolation and expansion

Peripheral blood was obtained from normal healthy adult donors after informed consent from the New York Blood Center and mononuclear cells were isolated by Ficoll-Paque (Amersham Biosciences, Piscataway, NJ, USA) density gradient separation as we have previously described [45].

NK cells were expanded in tissue culture plates with K562-mbIL15-41BBL in RPMI 10 IU/ml of IL-2 (R&D Systems, Inc., Minneapolis, MN, USA) and 10% FCS at 37°C with 5% CO2 as we have previously described [11, 46].

### Antibody dependent cell mediated cytotoxicity assay

ADCC was determined using DELFIA EuTDA cytotoxicity assay as we have previously described [45]. 1 × 10^6^ target cells were labeled with BATDA labeling ligand for 30 minutes. Tumor target cells were washed and incubated with 10 μg/ml obinutuzumab, rituximab or polyclonal IgG at 37°C and incubated with expanded NK effector cells, at 10:1 and 5:1 E: T target ratio for 4 hours at 37°C. Plates were centrifuged and the supernatant was collected into a flat-bottom plate and Europium solution was added and incubated for 15 minutes at room temperature. Fluorescence was measured with a time-resolved fluorometer (Perkin Elmer). The percentage of cytotoxicity was calculated using following equation: % specific release = (E-S/(M-S) × 100) E = experimental release, S = spontaneous release (target cells without effector cells), M = maximum release (lysed target cells).

### Gene expression microarray

Gene expression experiments were performed using Illumina Human-HT12 v4.0 array at the Rockefeller University Genomics Facility (New York, NY, USA). Biotin-labeled RNA was prepared using 200 μg of total RNA by MessageAmp™ Premier RNA Amplification Kit (Applied Biosystems, Foster City, CA, USA). Antisense RNA (750 ng) was mixed with hybridisation reagents and heated at 65°C for 5 minutes. After it was cooled to room temperature, hybridization solution was applied to Illumina HumanHT-12 v4 array. The array was scanned using Illumina BeadArray Reader. The scanning was done using standard DirectHyb Gene Expression protocol. The raw data was extracted using Illumina BeadStudio software without normalization. Data has been deposited in the Gene Expression Omnibus database (accession number GSE75605).

GSEA with MSigMB v5.2 software was used to investigate the biological processes and pathways differentially implicated between obinutuzumab treated and PBS or IgG treated PBML cells [47, 48]. Genes with > 1.5 fold or < 1.5 fold expression ratio were considered to be significantly differentially expressed genes as we and others have previously discussed [48, 49].

### *In-vivo* human PMBL xenograft NSG mice studies

The experimental animal protocols, procedures and care were approved by the Institutional Animal Care and Use Committee, at New York Medical College (NYMC, 33-2-0615H), Valhalla, NY, USA on January 20, 2015. Karpas-1106P PMBL cell line was stably transfected with a firefly luciferase expression plasmid (*ffluc-zeo*) to generate Karpas-1106P Luc+ cells [11]. Mice were γ- irradiated (2.5 Gy) 1 day before tumor cells were injected. Six- to eight- week-old female NSG (NOD. Cg-Prkdc^scid^ *Il2rg*^tm1Wjl^/SzJ) mice from Jackson laboratory (Bar Harbor, ME, USA) were intravenously injected with 5 × 10^6^ Karpas-1106P Luc+ cell as we have previously described [45].

The *in-vivo* anti-tumor function of obinutuzumab, (30 mg/kg) and rituximab (30 mg/kg) was determined in 6–8 week old mice. NSG mice were divided into 4 groups: 1) PBS only (control), 2) isotype control (IgG), 3) obinutuzumab (30 mg/kg), and 4) rituximab (30 mg/kg). One week after tumor cell injection, mice were injected every 7 days with the respective therapy for 8 weeks. Tumor regression and/or progression of xenografted mice were monitored weekly by *in-vivo* bioluminescence imaging and survival was monitored for 250 days.

### Statistical analysis

Data obtained from 3 independent experiments are represented as means ± SD and statistical significance of the difference between two groups was determined by using two-tailed Student’s *t*-test. In *in-vivo* study, survival rates were analyzed by the Kaplan-Meier method and their statistical significance was evaluated by log-rank test using the Prism Version 6.0 software. *P* values < 0.05 were considered statistically significant for all *in-vitro* and *in-vivo* studies.

## SUPPLEMENTARY MATERIALS



## Abbreviations

PMBL: primary mediastinal large B-cell lymphoma
B-NHL: B-non Hodgkin lymphoma
BL: Burkitt lymphoma
JAK: Janus kinase
STAT: signal transducer and transcription
ADCC: antibody dependent cellular cytoxicity
FL: follicular lymphoma
NK: natural killer
PBS: phosphate buffer saline
GSEA: gene set enrichment analysis
DLBCL: diffuse large B-Cell lymphoma
