# Supplementary material for: Obinutuzumab (GA101) vs. rituximab significantly enhances cell death, antibody-dependent cytotoxicity and improves overall survival against CD20+ primary mediastinal B-cell lymphoma (PMBL) in a xenograft NOD-scid IL2Rgnull (NSG) mouse model: a potential targeted agent in the treatment of PMBL
Source: Oncotarget. 2020 Aug 11;11(32):3035–47. doi: 10.18632/oncotarget.27691 (PMC7429176; doi:10.18632/oncotarget.27691)
Supplement: Supplementary file 1 [file oncotarget-11-3035-s001.pdf]

Obinutuzumab (GA101) vs. rituximab significantly enhances cell death, antibody-dependent cytotoxicity and improves overall survival against CD20+ primary mediastinal B-cell lymphoma (PMBL) in a xenograft NOD-scid IL2Rgnull (NSG) mouse model: a potential targeted agent in the treatment of PMBL

SUPPLEMENTARY MATERIALS

Supplementary Table 1: Hallmark gene sets ($p < 0.05$ and FDR < 0.1) between obinutuzumab and IgG treatments

TERM	NES	*P*	FDR
HALLMARK_E2F_TARGETS	–6.32	0	0
HALLMARK_G2M_CHECKPOINT	–5.38	0	0
HALLMARK_DNA_REPAIR	–3.51	0	0
HALLMARK_MITOTIC_SPINDLE	–3.33	0	0
HALLMARK_INTERFERON_ALPHA_RESPONSE	–2.67	0	0.0001
HALLMARK_PROTEIN_SECRETION	–2.20	0.0018	0.0036
HALLMARK_MYC_TARGETS_V1	–1.77	0.0188	0.0448
HALLMARK_PI3K_AKT_MTOR_SIGNALING	–1.64	0.0278	0.0764
HALLMARK_REACTIVE_OXIGEN_SPECIES_PATHWAY	1.55	0.0347	0.0819
HALLMARK_EPITHELIAL_MESENCHYMAL_TRANSITION	1.67	0.0318	0.0509
HALLMARK_KRAS_SIGNALING_UP	1.90	0.0124	0.0161
HALLMARK_IL6_JAK_STAT3_SIGNALING	1.91	0.0095	0.0162
HALLMARK_ESTROGEN_RESPONSE_LATE	1.98	0.0051	0.0104
HALLMARK_HEDGEHOG_SIGNALING	1.99	0.0042	0.0105
HALLMARK_INTERFERON_GAMMA_RESPONSE	2.09	0	0.0054
HALLMARK_NOTCH_SIGNALING	2.14	0	0.0041
HALLMARK_ESTROGEN_RESPONSE_EARLY	2.14	0	0.0044
HALLMARK_P53_PATHWAY	2.16	0	0.0043
HALLMARK_MYC_TARGETS_V2	2.20	0	0.0032
HALLMARK_UV_RESPONSE_UP	2.47	0	0.0006
HALLMARK_APOPTOSIS	2.49	0	0.0002
HALLMARK_MTORC1_SIGNALING	2.74	0	0
HALLMARK_INFLAMMATORY_RESPONSE	2.78	0	0
HALLMARK_IL2_STAT5_SIGNALING	3.12	0	0
HALLMARK_GLYCOLYSIS	3.52	0	0
HALLMARK_TNFA_SIGNALING_VIA_NFKB	5.04	0	0
HALLMARK_HYPOXIA	5.66	0	0

FDR: false discovery rate; NES: normalized enrichment score.

Supplementary Table 2: Hallmark gene sets ($p < 0.05$ and FDR < 0.1) between obinutuzumab and PBS treatments

TERM	NES	*P*	FDR
HALLMARK_INTERFERON_ALPHA_RESPONSE	–5.36	0	0
HALLMARK_E2F_TARGETS	–4.84	0	0
HALLMARK_G2M_CHECKPOINT	–4.24	0	0
HALLMARK_INTERFERON_GAMMA_RESPONSE	–3.69	0	0
HALLMARK_MITOTIC_SPINDLE	–3.22	0	0
HALLMARK_DNA_REPAIR	–3.04	0	0
HALLMARK_ALLOGRAFT_REJECTION	–2.03	0.0092	0.0078
HALLMARK_PANCREAS_BETA_CELLS	–1.84	0.0116	0.0225
HALLMARK_PROTEIN_SECRETION	–1.70	0.0372	0.0434
HALLMARK_APICAL_SURFACE	1.50	0.0426	0.0938
HALLMARK_KRAS_SIGNALING_UP	1.57	0.0431	0.0754
HALLMARK_NOTCH_SIGNALING	1.75	0.0144	0.0304
HALLMARK_REACTIVE_OXIGEN_SPECIES_PATHWAY	1.85	0.0212	0.0205
HALLMARK_UV_RESPONSE_DN	1.88	0.0087	0.0186
HALLMARK_EPITHELIAL_MESENCHYMAL_TRANSITION	1.99	0.0096	0.0096
HALLMARK_UNFOLDED_PROTEIN_RESPONSE	2.05	0.0028	0.0073
HALLMARK_WNT_BETA_CATENIN_SIGNALING	2.06	0.0071	0.0074
HALLMARK_ESTROGEN_RESPONSE_LATE	2.18	0.0033	0.0041
HALLMARK_HEDGEHOG_SIGNALING	2.21	0	0.0039
HALLMARK_P53_PATHWAY	2.24	0	0.0037
HALLMARK_IL6_JAK_STAT3_SIGNALING	2.40	0	0.0015
HALLMARK_UV_RESPONSE_UP	2.55	0	0.0004
HALLMARK_MYC_TARGETS_V2	2.69	0	0
HALLMARK_ESTROGEN_RESPONSE_EARLY	2.77	0	0
HALLMARK_MTORC1_SIGNALING	2.95	0	0
HALLMARK_APOPTOSIS	2.96	0	0
HALLMARK_IL2_STAT5_SIGNALING	3.06	0	0
HALLMARK_GLYCOLYSIS	3.32	0	0
HALLMARK_INFLAMMATORY_RESPONSE	3.36	0	0
HALLMARK_TNFA_SIGNALING_VIA_NFKB	4.89	0	0
HALLMARK_HYPOXIA	5.76	0	0

FDR: false discovery rate; NES: normalized enrichment score.
